# Predictive Value of C-Reactive Protein for Major Complications after Major Abdominal Surgery: A Systematic Review and Pooled-Analysis

**DOI:** 10.1371/journal.pone.0132995

**Published:** 2015-07-15

**Authors:** Jennifer Straatman, Annelieke M. K. Harmsen, Miguel A. Cuesta, Johannes Berkhof, Elise P. Jansma, Donald L. van der Peet

**Affiliations:** 1 Department of Gastrointestinal Surgery, VU University Medical Center, Amsterdam, the Netherlands; 2 Department of Epidemiology and Biostatistics, VU University Medical Center, Amsterdam, the Netherlands; 3 Medical Library, VU University Medical Center, Amsterdam, the Netherlands; The Chinese University of Hong Kong, HONG KONG

## Abstract

**Background:**

Early diagnosis and treatment of complications after major abdominal surgery can decrease associated morbidity and mortality. Postoperative CRP levels have shown a strong correlation with complications. Aim of this systematic review and pooled-analysis was to assess postoperative values of CRP as a marker for major complications and construct a prediction model.

**Study design:**

A systematic review was performed for CRP levels as a predictor for complications after major abdominal surgery (MAS). Raw data was obtained from seven studies, including 1427 patients. A logit regression model assessed the probability of major complications as a function of CRP levels on the third postoperative day. Two practical cut-offs are proposed: an optimal cut-off for safe discharge in a fast track protocol and another for early identification of patients with increased risk for major complications.

**Results:**

A prediction model was calculated for major complications as a function of CRP levels on the third postoperative day. Based on the model several cut-offs for CRP are proposed. For instance, a two cut-off system may be applied, consisting of a safe discharge criterion with CRP levels below 75 mg/L, with a negative predictive value of 97.2%. A second cut-off is set at 215 mg/L (probability 20%) and serves as a predictor of complications, indicating additional CT-scan imaging.

**Conclusions:**

The present study provides insight in the interpretation of CRP levels after major abdominal surgery, proposing a prediction model for major complications as a function of CRP on postoperative day 3. Cut-offs for CRP may be implemented for safe early-discharge in a fast-track protocol and, secondly as a threshold for additional examinations, such as CT-scan imaging, even in absence of clinical signs, to confirm or exclude major complications. The prediction model allows for setting a cut-off at the discretion of individual surgeons or surgical departments.

## Introduction

Twenty percent of patients after major abdominal surgery (MAS) have a major postoperative complication, which requires invasive treatment and is associated with increased morbidity and mortality [1, 2].

Early diagnosis and treatment of major complications is associated with improved outcomes [3, 4]. However, early detection may be challenging and can be difficult to distinguish from the physiological postoperative systemic inflammatory response syndrome (SIRS) [5, 6]. Major complications, such as anastomotic leakage, may be clinically silent and not become evident until critical illness develops after a median of seven days [7].

A standardized postoperative quality-control algorithm for risk assessment of complications should aim at early identification of patients for a safe early discharge—as in a fast-track protocol—or help to identify patients at risk of developing major complications. This policy could decrease associated morbidity and mortality [8].

Several examinations contributed to the development of such a postoperative quality control algorithm. Postoperatively, clinical parameters, laboratory markers and imaging all contribute to identification of complications. Clinical parameters have shown to be non-specific, especially in the early postoperative phase, warranting additional examinations [1]. The acute phase protein CRP (C-reactive protein) has shown to have a strong correlation with postoperative complications [9, 10]. Postoperative CRP levels rise in response to the initial surgery in order to peak 48–72 hours postoperatively and decrease thereafter [11]. In patients with a complicated postoperative course, postoperative CRP levels remain elevated [9, 12].

Many studies aimed to establish a cut-off value for CRP as a predictor for postoperative complications, such as anastomotic leakage or septic complications [10, 13–17]. Results are however conflicting, postoperative complications are not stratified and most studies merely aimed to set a cut-off for CRP as a marker for anastomotic leakage after colorectal surgery [10, 13, 16, 17].

With such a variance in cut-offs and definitions, it would be of interest to assess the predictive value of CRP levels as a continuous value. This would enable assessment of separate CRP levels, allowing surgical wards to set a cut-off for additional examinations or safe discharge at their own discretion. Aim of this study was to conduct a systematic review and pooled-analysis of literature assessing the prediction of major complications as a function of continuous levels of CRP.

## Methods

This systematic review was conducted in accordance with the Preferred Reporting Items for Systematic Reviews and Meta-Analysis (PRISMA statement) [18].

### Definitions

All complications were stratified according to the Clavien-Dindo (CD) classification, which grades complications according to the required treatment [19, 20]. In line with recent literature, we further divided the CD classification into two groups, minor and major complications respectively [21]. Minor complications consist of grade I and grade II complications, which require no treatment or pharmacological treatment. Wound infections drained at the bedside are also considered minor complications. Major complications consist of CD grades III and up. These complications require surgical, endoscopic or radiological intervention and might lead to Intensive Care Unit admission or death. These complications include for instance reoperation for anastomotic leak or percutaneous drainage of abscesses.

### Data sources and searches

Systematic electronic searches were conducted in the bibliographic database MEDLINE, Embase and the Cochrane Library (via Wiley) from inception to August 2014, in collaboration with a medical librarian. Search terms included controlled terms from MeSH as well as free text terms in PubMed, EMtree in Embase. We used free text terms only in The Cochrane library. An additional manual-check of article references was done in order to identify potential records of interest. The search strategy was altered for Embase and the Cochrane Library databases. We applied a language restriction; only English, German and Dutch articles were included. The search was further limited to “clinical studies” and articles with an abstract. The separate results from all searches were reconciled for duplicate articles. The abstracts obtained by the search in the three databases were combined and were used to select suitable articles by two reviewers independently (J.S. and A.H.), after which the full-text versions were retrieved and independently reviewed for inclusion by the two reviewers (J.S. and A.H.). Investigators with previous experience in reviews conducted the systematic review (A.H., E.J. and J.S.).

### Inclusion and exclusion

Studies reporting on analyzing the diagnostic accuracy of the serum CRP as marker for major postoperative complications after MAS, which we defined as a surgical resection performed on upper-gastrointestinal, hepato-pancreatico-biliary and colorectal organs, as carried out by either primary anastomosis and/or creation of definitive stoma, were included. For instance, cholecystectomy is not included, since no anastomosis or ostomy is performed. Previous work has shown no statistically significant differences in postoperative CRP levels after upper-gastrointestinal, hepato-pancreatico-biliary and colorectal surgery [1].

The studies had to provide adequate information on the time of CRP measurement, the outcome and unit in which it was measured and also postoperative complications. Sensitivity, specificity, positive and negative predictive values, and the area under the curve had to be measured using logistic regression analysis (ROC curve). Exclusion criteria were: 1) duplicate studies of previously published data; 2) data pooled from several different postoperative days (POD); 3) no clear delineation provided of the time of CRP measurement; 4) postoperative complications that consisted only of minor surgical site infections (SSI); and 5) pediatric studies.

### Quality Assessment

The quality of the studies was appraised using the Quality Assessment of Diagnostic Accuracy Studies (QUADAS) criteria [22]. The QUADAS-tool assesses the internal and external validity of diagnostic accuracy studies, by reviewing the quality of the studies, the risk of bias and the concerns regarding applicability. Two reviewers (J.S. and A.H.) independently applied the QUADAS-tool to all the studies included in the pooled-analysis for making an overall risk-of-bias judgment.

### Data extraction and statistical analysis

Raw data was collected and pooled ROC-analysis was performed. True-/false positive, true-/false negative, sensitivity, specificity, area under the curve (AUC), positive-/ negative predictive and true cut-off values were calculated for each individual study. Data on true-/false positive, true-/false negative, sensitivity, specificity and the AUC were calculated. The cut-off of the pooled data was determined by maximizing the sum of the sensitivity and specificity (Youden index) [23]. Heterogeneity with respect to sensitivity and specificity was tested by a likelihood-ratio test for the variance component in a random-effects model. Positive predictive value (PPV) and negative predictive value (NPV) are study dependent and can therefore not be pooled. The risk of major complications was predicted from continuous CRP values by a logit regression model [24]. Reported ninety-five percent confidence intervals were obtained via Wald’s method [25].

Diagnostic accuracy for CRP as marker for major complications was calculated by ROC-analysis, maximizing the sum of the sensitivity and specificity (Youden-index). With the highest-measured accuracy for CRP on POD 3, a logit regression model of the probability of major complications as a function of continuous CRP levels was calculated. Based on this regression model, two practical cut-offs were calculated: a) the first as a marker with a high negative predictive value, which could be used for safe early discharge in a fast-track protocol; and b) a marker with a high positive predictive value, to identify patients in whom a CT-scan is strictly indicated for confirming or excluding a major postoperative complication.

## Results

**Fig 1:**
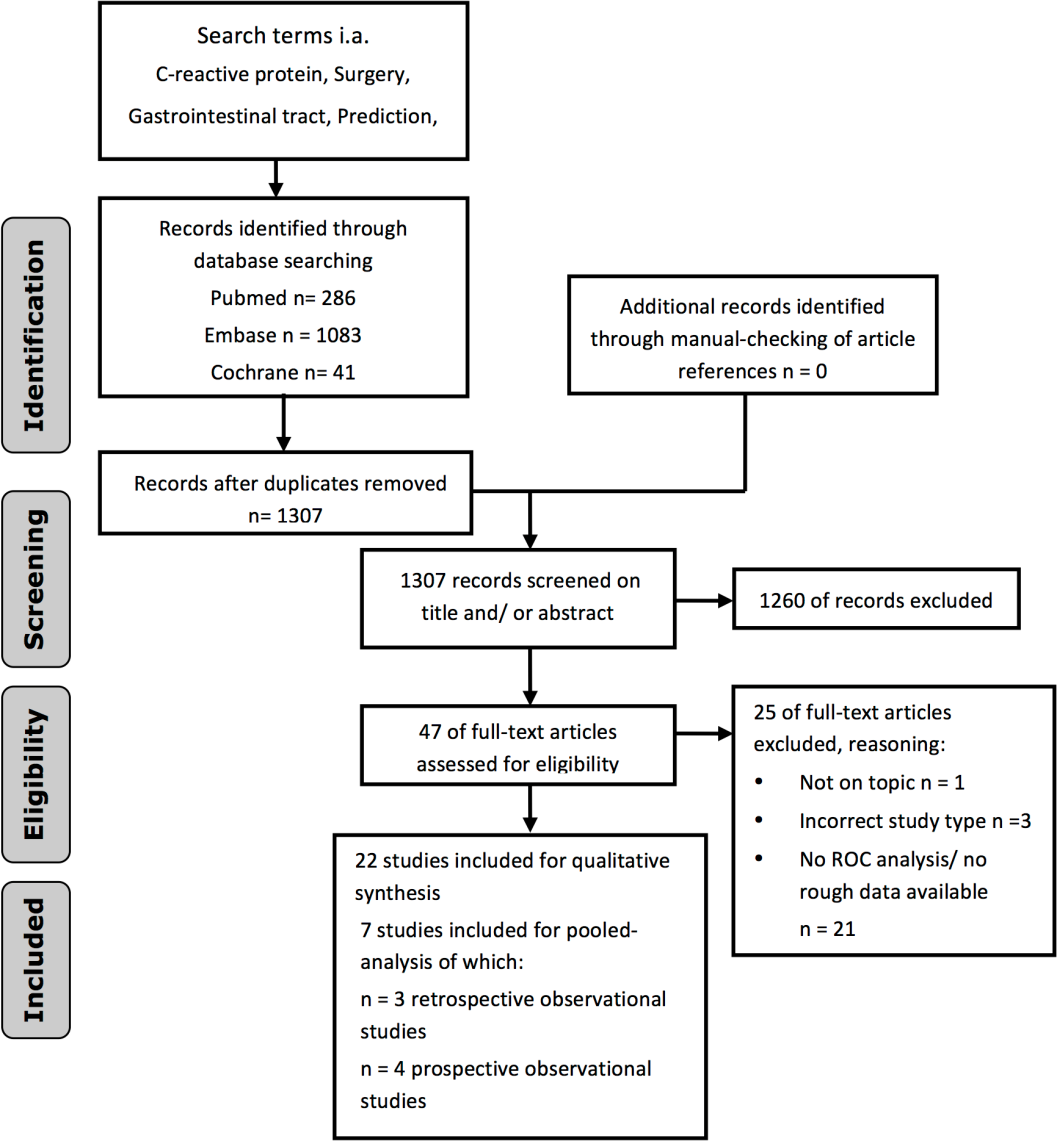
Flow chart for literature search.

A total of 1307 articles were initially identified and screened. A flow-chart for the literature search is depicted in Fig 1. Based on abstract and title, 1260 articles were discarded. Forty-six articles were assessed in full-text format, whereas 25 articles were excluded because of different reasons as depicted in the flowchart of Fig 1. Finally a total of 22 studies were identified that stated an optimal cut-off value for CRP as marker for safe discharge, or as a marker for major postoperative complications or a certain type of complication, such as anastomotic leak. These articles are summarized in Table 1. Thirteen studies proposed a cut-off for CRP as a marker for postoperative complications [1, 10, 12–15, 17, 26–31]. Definitions varied widely, ranging from one study including all complications, to another study including only postoperative sepsis. Nine studies proposed a cut-off for CRP as a marker for anastomotic leakage [9, 16, 32–38]. Again, definitions of leakage varied widely, ranging from studies including all patients with clinical signs of leakage to studies including only patients with a leak confirmed by imaging or upon reoperation. Table 1 portrays an overview of definitions of complications used.

**Table 1:** Overview of definitions used for complications and anastomotic leakage in the selected studies. Abreviations; postoperative day (POD), anastomotic leak (AL), systemic inflammatory response syndrome (SIRS), computed tomography scan (CT), urinary tract infection (UTI).

**CRP as marker for postoperative complications**					
Study	**year**	**Organ**	**Cut-off**	**POD**	**Definition**
Adamina [26]	2014	Colorectal	56 mg/L	4	Infectious complications, graded according to Clavien-Dindo. Cut-off applies in absence of clinical signs.
Kørner [27]	2009	Colorectal	190 mg/L	3	Intraabdominal infection; AL, abscess or diffuse peritonitis
Lane [28]	2012	Colorectal	150 mg/L	2	Adverse events: including infective complications, postoperative organ dysfunction and prolonged length of stay
MacKay [15]	2009	Colorectal	145 mg/L	4	All infective complications
Mokart [29]	2005	All abdominal	93 mg/L	1	Postoperative sepsis (SIRS + infection)
Nason [30]	2014	Colorectal	148 mg/L	4	Infective complications; AL confirmed by CT, wound infection with purulent drainage
Platt [14]	2012	Colorectal	170 mg/L	3	Postoperative infective complications (surgical site and remote site infection)
Straatman [1]	2014	Major abdominal surgery	145 mg/L	3	Postoperative complications defined by Clavien-Dindo, with a cut-off for major complications (grades 3 and up)
Warschkow [31]	2012	Pancreas	94 mg/L	7	Postoperative inflammatory complications; pancreatic fistula, anastomotic leak, cholangitis, pancreatitis, wound infections, abscesses, pneumonia, UTI
Warschkow [13]	2012	Gastro-esophageal	141 mg/L	4	Postoperative infections; AL, abscess, pneumonia, wound infection, UTI, colitis
Warschkow [17]	2012	Colorectal	135 mg/L	4	Postoperative infectious complications: Any septic event, both intra- and extra- abdominal infections
Warschkow [10]	2011	Colorectal	123 mg/L	4	Inflammatory complications; AL (confirmed by imaging or operation), UTI, wound infection, pneumonia, central line infections.
Welsch [12]	2008	Pancreas	140 mg/L	4	Fistula, leak, abscess, wound infection, pneumonia, cholangitis, central line infection, UTI, necrotizing pancreatitis, infectious bilioma or pleural effusion
**CRP as marker for anastomotic leakage (AL)**					
**Study**	**year**	**Organ**	**Cut-off**	**POD**	**Definition**
Almeida [32]	2012	Colorectal	140 mg/L	3	AL defined as free feacal fluid in the abdomen diagnosed by drain production or CT-scan imaging
Deitmar [33]	2009	Oesophagus	135 mg/L	2	Leak of anastomosis or gastric staple line confirmed with endoscopy
Dutta [34]	2011	Esophago-gastric	180 mg/L	3	AL confirmed with CT, contrast study or upor reoperation
Garcia-Granero [35]	2013	Colorectal	147 mg/L	3	AL confirmed with imaging or upon reoperation
Oberhofer [36]	2012	Colorectal	99 mg/L	3	AL (imaging), abscess, wound infection, pneumonia, central line infection, UTI
Ortega- Deballon [16]	2010	Colorectal	125 mg/L	4	AL: feacal drain production, collection at anastomosis site with imaging, dehiscence during reoperation
Pedersen [37]	2012	Colorectal (MIS)	200 mg/L	3	AL diagnosed in patients with acute abdomen, upon imaging or upon reoperation
Scepanovic [38]	2013	Stomach, small bowel, colon	135 mg/L	3	AL defined as clinical presence of enteric contents within the drains
Welsch [9]	2007	Rectal	140 mg/L	3&4	AL (imaging), abscess, wound infection, pneumonia, central line infection, UTI

All authors of articles selected were contacted via e-mail or telephone and asked to provide raw data regarding collected CRP values and complications as graded according to the Clavien-Dindo classification in their studies in order to conduct a pooled analysis of the raw data. Authors of 11 studies never replied, of two studies no contact details were provided and the authors of two studies replied, stating they had no time or interest to participate. Seven authors provided complete raw data [1, 27, 32, 35, 37–39]. All authors reassessed their data regarding all complications using the Clavien-Dindo classification. These seven studies were included for pooled-analysis comprising a total of 1427 patients. The characteristics of the seven studies are provided in Table 2. Four studies had a prospective design. All seven studies included both malignant and benign indications for surgical intervention. CRP values were available in six studies on POD 3, in five studies on POD 4 and in six studies on POD 5.

**Table 2:** Characteristics of the studies included for pooled-analysis. Abbreviations: months (mo), minimally invasive surgery (MIS).

Study	Design	Study interval	Operation	Indication (acute/elective)	n	Major Complication
Almeida et at. (2012) [32]	Retrospective	22 mo	Colorectal resection	Both	173	21 of 173 (12.1%)
Garcia-Granero et al. (2013)[35]	Prospective	17 mo	Colorectal resection	Both	205	17 of 205 (8.3%)
Kørner et al. (2009) [27]	Retrospective	12 mo	Colorectal resection	Both	231	23 of 231 (10%)
Lagoutte et al. (2012) [39]	Retrospective	14 mo	Colorectal resection	Both	100	20 of 100 (20%)
Pedersenet al. (2012) [37]	Retrospective	12 mo	MIS Colorectal resection	Both	163	41 of 163 (25.2%)
Scepanovic et al. (2013) [38]	Prospective	18 mo	Al digestive resections with anastomosis	Both	156	15 of 156 (9.6%)
Straatman et al. (2014) [1]	Retrospective	24 mo	All digestive resections with anastomosis/ostomy	Both	399	82 of 399 (20.6%)

### Risk of bias

The methodological quality of the included studies varied. There were five studies on colorectal surgery and two including all digestive tract operations. There was considerable variation in the quality of the included studies as depicted in Table 3. Two items were scored as “no” for each of the included studies, namely the uninterpretable/intermediate test results were not reported or if withdrawals from the study were explained, as they were not relevant and applicable. Only five items were included in all studies. For the remaining eight items, it was of special concern that high numbers of the studies were not reported if patients received the same reference standard regardless of the index test result and if the reference standard was independent of the index test.

**Table 3:** The QUADAS tool for classification of accuracy of the studies selected from literature, according to number of studies in each response category.

QUADAS criteria	Number of articles in each category		
	Yes No Unclear		
Was the spectrum of patients representative of the patients who will receive the test in practice?	7	-	-
Were selection criteria clearly described?	7	-	-
Is the reference standard likely to correctly classify the target condition? RS: x, CT-scan of diag lap	7	-	-
Is the time period between reference standard and index test short enough to be reasonably sure that the target condition did not change between the two tests?	7	-	-
Did the whole sample or a random selection of the sample, receive verification using a reference standard of diagnosis?	-	7	-
Did patients receive the same reference standard regardless of the index test result?	2	5	-
Was the reference standard independent of the index test (i.e. the index test did not form part of the reference standard)?	1	6	-
Was the execution of the index test described in sufficient detail to permit replication of the test?	4	3	-
Was the execution of the reference standard described in sufficient detail to permit its replication?	6	1	-
Were the index test results interpreted without knowledge of the results of the reference standard?	5	1	1
Were the reference standard results interpreted without knowledge of the results of the index test?		7	
Were the same clinical data available when test results were interpreted as would be available when the test is used in practice?	7		
Were uninterpretable/ intermediate test results reported?	1	5	1
Were withdrawals from the study explained?	2	4	1

### CRP prediction model

Raw data from the seven studies was pooled and analyzed. Major complications, as classified according to the Clavien-Dindo grades 3, 4 and 5, occurred in an average 15.4% of patients (range 8.3–25.2%). Average CRP levels in patients with major complications and patients with no or minor complications for postoperative days 3, 4 and 5 are depicted in Table 4.

**Table 4:** Median CRP levels and interquartile ranges (IQR) for each postoperative day in the included studies for patients with major complications versus patients with an uncomplicated course or minor complication. NA = Not Available

Study	CRP POD 3		CRP POD 4		CRP POD 5	
	Uncomplicated or minor complication	Major complication	Uncomplicated or minor complication	Major complication	Uncomplicated or minor complication	Major complication
Almeida [32]	99 (78–122)	167 (127–307)	58 (37–93)	179 (117–237)	28 (22–35)	124 (104–262)
Garcia-Granero [35]	125 (71–186)	255 (188–270)	85 (50–138)	260 (120–278)	56 (31–100)	248 (137–285)
Korner [27]	113 (69–199)	256 (180–317)	NA	NA	54 (30–117)	193 (95–313)
Lagoutte [39]	112 (83–170)	227 (140–270)	80 (50–127)	160 (100–284)	NA	NA
Pedersen [37]	NA	NA	NA	NA	113 (69–223)	194 (125–289)
Scepanovic [38]	114 (87–140)	168 (113–195)	79 (62–113)	145 (84–190)	57 (39–98)	127 (61–157)
Straatman [1]	157 (108–229)	245 (163–331)	124 (82–171)	189 (125–296)	94 (54–172)	159 (89–253)

**Fig 2:**
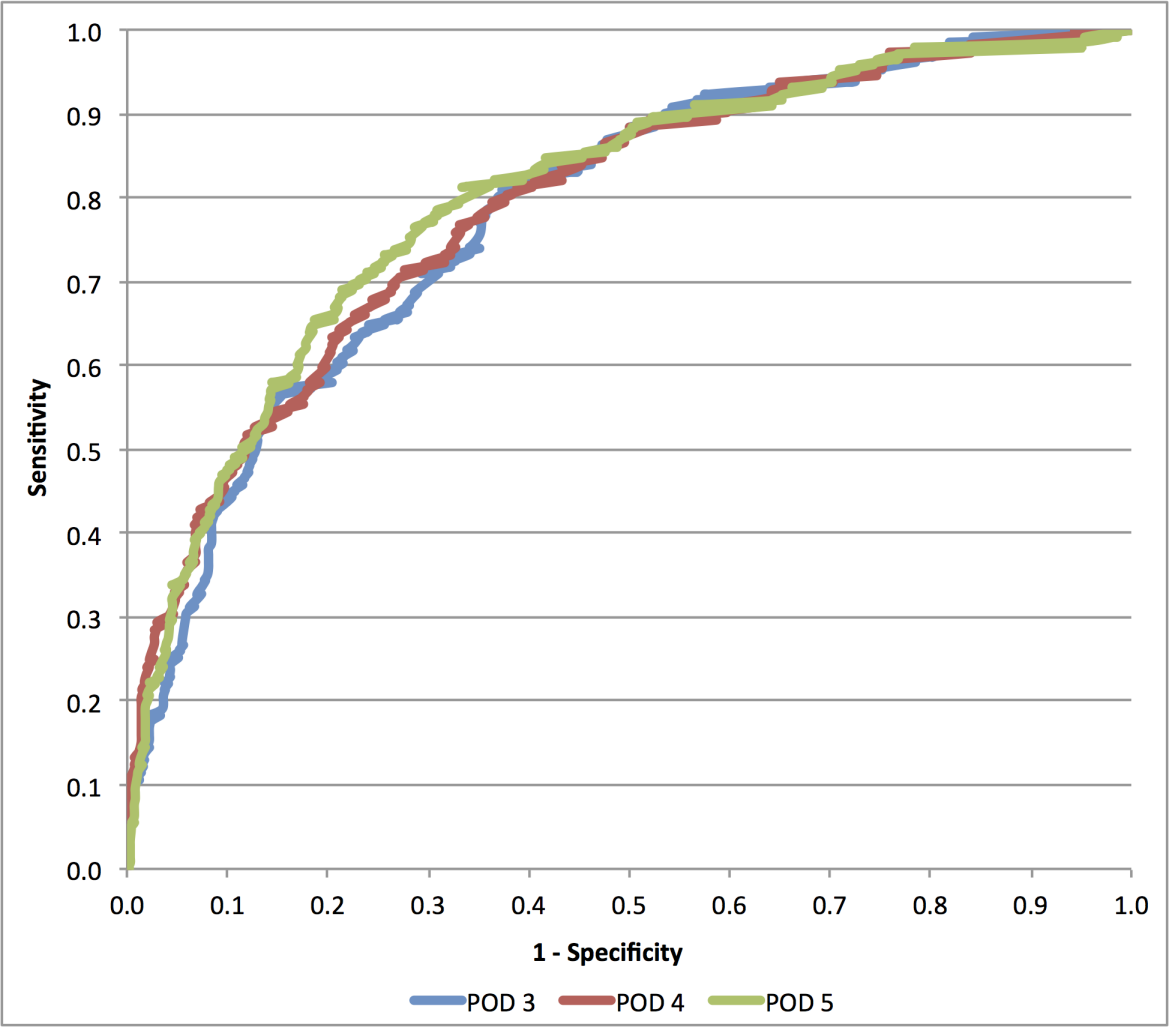
Receiver Operator Characteristics (ROC) curve for CRP levels on postoperative day (POD) 3,4 and 5.

First pooled-analysis with receiver operator characteristic (ROC) curve analysis was used to assess the highest sensitivity and AUC for CRP as a marker of major complications, as depicted in Fig 2. According sensitivity and specificity are depicted in Table 5. With the highest measured sensitivity for CRP on POD 3, a logit regression model of the probability of major complications as a function of continuous CRP levels was calculated for postoperative day 3.

**Table 5:** Receiver Operator Characteristics (ROC) curve analysis for pooled data with area under the curve (AUC), positive predictive values(PPV) and negative predictive values (NPV) for each postoperative day (POD). Positive and negative likelihood ratios (LR+ and LR-) were calculated to assess the difference in odds of complications pre- and post CRP measurement.

POD	cut-off	AUC	95% CI	Sensitivity	Specificity	PPV	NPV	LR+	LR-
POD 3	140	0.783	0.742–0.824	81.7%	61.6%	25.7%	95.4%	2.13	0.30
POD 4	130	0.79	0.744–0.836	71.4%	72.5%	31.6%	93.4%	2.60	0.39
POD 5	101	0.800	0.740–0.840	76.6%	71.0%	34.9%	93.7%	2.64	0.33

**Fig 3:**
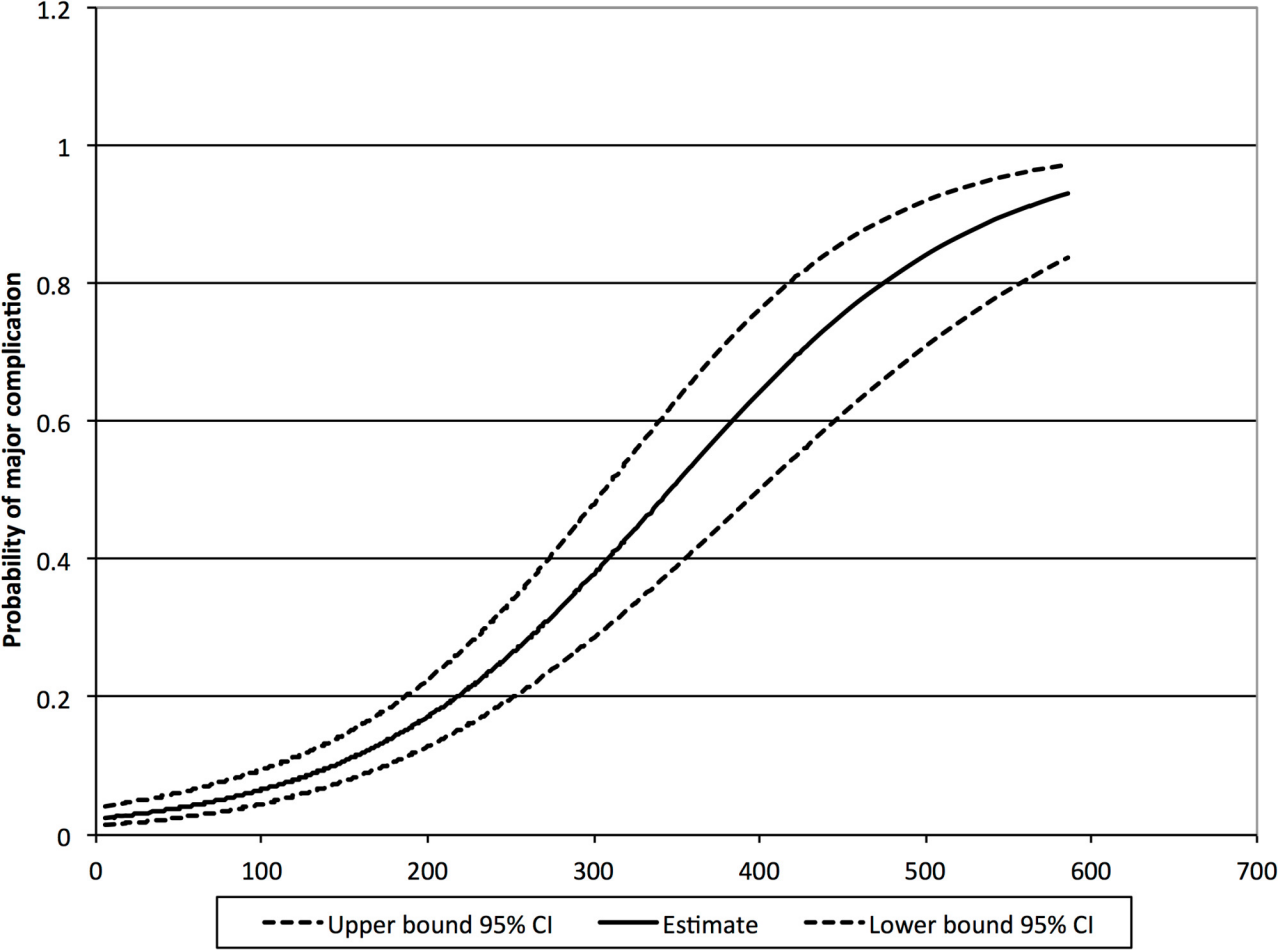
Prediction for the probability of major complications as a function of measured CRP levels on postoperative day three, with 95% confidence interval. Depicts probability of complications for individual CRP measurements.

The prediction model is depicted in Fig 3 and Table 6 with according 95% confidence intervals. Following this risk assessment, cut-offs may be established at the discretion of the surgeon or surgical department. For instance an optimal cut-off was observed at 140 mg/L, with a sensitivity of 81.7% and specificity of 61.6%. One may also wish to establish two practical cut-offs. First, a cut-off for early safe discharge with a predictive value below 5% (95% CI: 3.3–7.5%) is calculated at 75 mg/L on POD 3 with a sensitivity of 96.2%, specificity of 21.7%, positive predictive value of 16.6% and a negative predictive value of 97.2%. Second, a cut-off that can be used as an identifier of those patients at high risk of developing major complications thereby indicating additional examinations; demonstrating a predictive value for CRP above 20% (95% CI: 14.7–25.6%). The latter cut-off was calculated at 215 mg/L for POD 3, with a sensitivity of 57.3%, specificity of 82.8%, positive predictive value of 35% and negative predictive value of 92.5%.

**Table 6:** Probability of major complications for different CRP levels, with the probability of the upper cut-off of 215 mg/L depicted.

CRP level	Probability	95% confidence interval	
mg/L	%	Lower	Upper
50	3.94%	2.51%	6.13%
**75**	**5%**	**3.33%**	**7.55%**
100	6.51%	4.46%	9.39%
150	10.77%	7.83%	14.64%
200	17.16%	12.85%	22.53%
**215**	**20.00%**	**14.70%**	**25.60%**
250	26.42%	19.96%	34.10%
300	37.88%	28.63%	48.10%
350	52.48%	40.07%	64.59%
400	62.98%	48.98%	75.09%

## Discussion

The presented systematic review and pooled-analysis, assesses the role of CRP as a marker for major complications.

Different studies have assessed CRP as a marker for complications and aimed to set a cut-off. Twenty-two studies were identified with cut-offs for CRP ranging from 140–190 mg/L on postoperative day three and 54–148 mg/L on postoperative day four. The wide range in cut-offs can be explained by the variety in definitions for complications depicted in the studies. Implementation of the Clavien-Dindo classification for complications allows for more reproducible assessment of complications [19].

The logistic regression model predicts major complications following major abdominal surgery as a function of continuous CRP levels on postoperative day three. The prediction models allows for assessment of separate CRP values, but also enables surgical wards to establish a cut-off for CRP at their own discretion. This cut-off can be established to differentiate between those patients at risk of developing major complications and those patients with an uncomplicated postoperative course or minor complications.

The positive predictive value of this practical cut-off may seem low, indicating that CRP levels also may be high in patients with a prolonged systemic inflammatory response syndrome based on severity of surgical trauma and not due to major complications. Yet it will most certainly help to identify major complications in an early phase in more than one-third of patients after MAS [15]. If clinical symptoms are not clear, determining whether CRP levels are decreasing or increasing in the following days can provide indications whether patients can be discharged safely or that a CT-scan has to be performed.

Positive predictive values remain low in all studies, further demonstrating that a high CRP-level alone is not sufficient for diagnosis of major complications but serves as an important indicative.

Given that CRP is non-specific for location or type of complication in combination with the low positive predictive value, it warrants additional examination. Computed Tomography (CT) is currently the most readily available imaging in the work-up of major complications [5, 40, 41]. Several studies have assessed the role of CT-scan imaging in diagnosis of major complications such as anastomotic leaks and found a sensitivity of 97%, therefore it is considered to be the imaging modality of choice [42, 43].

The prediction model allows for establishment of a cut-off at the discretion of the surgeon. A statistically optimal cut-off on POD 3 of 140 mg/L was calculated as an initial marker to identify major complications after MAS, with a sensitivity of 81.7% and specificity of 61.6%. Diagnostic accuracy for CRP was similar on postoperative day 3, 4 and 5 [3, 4, 44]. The results are in concordance with recent literature [14, 26, 44]. The relatively low sensitivity of 81.7% and a positive predictive value of 25.6% imply that a CT-scan would be performed whilst only one in four patients will be diagnosed with a major complication.

A more practical example would entail a double cut-off system. The first cut-off should correspond with a high negative predictive value in order to ensure early safe discharge in a fast track protocol. The second cut-off should aim at identifying patients with a high probability (a.s. >20%) of major complications, indicating whether additional examinations are necessary. Using the logit regression model for the probability of major complications calculated as a function of continuous CRP levels we were able to assess the two cut-offs. The first cut-off, with a value of CRP of 75 mg/L has a negative predictive value of 97.2%, indicating that patients with CRP levels below this cut-off may be discharged safely [26]. The second cut-off was set at 215 mg/L as predictor for complications.

Differences between the included studies in study design, methodology and patient population cause the pooled-analysis to be limited by its heterogeneity. Three of the seven included studies had a retrospective design [1, 27, 37]; this impacts the selection bias as well as the information bias. One study had data available for all patients on each day, suggesting selective measurement of CRP levels in the other studies [38]. Five out of the seven studies for pooled analysis included only colorectal surgery, compared to all digestive resections in the latter two studies. All were included since previous studies have shown no statistically significant differences in CRP levels between the different operated organ groups [1]. Only 7 out of 22 contacted authors provided data, possibly creating an inclusion bias. All types of surgery and organ groups are equally included in the 7 studies that provided data. Blinding was not performed in any of the studies. By requesting raw data for CRP levels and all complications being re-graded according to the Clavien-Dindo classification, the risk-of-bias by differences in criteria for complications is diminished [19, 20]. The Clavien-Dindo classification does not provide information on the type and localization of complications.

The present study provides insight in the interpretation of CRP levels measured after major abdominal surgery, proposing a logit regression model of a pooled analysis of seven studies, one for safe early-discharge in a fast-track protocol. The other concerning a reasonable high positive predictive value in which a CT-scan, independently of clinical symptoms, will confirm or exclude a major complication. The prediction model provides further insight and allows for setting a cut-off at the discretion of individual surgeons.

Definitive results should be obtained in a prospective manner. A prospective randomized trial is currently in progress, in order to assess the role of standardized measurements of CRP in a step-up quality-control algorithm using as primary endpoint a decrease in morbidity and mortality after major abdominal surgery.

## Supporting Information

S1 Checklist A checklist displaying which PRISMA items are described on what page of the manuscript. (PDF)

S1 Appendix Search strategy. The document displays our search strategy for the Medline database. The search was adapted accordingly for the Embase and Cochrane databases
(DOCX)
