# Supplementary material for: Predictive Value of C-Reactive Protein for Major Complications after Major Abdominal Surgery: A Systematic Review and Pooled-Analysis
Source: PLoS One. 2015 Jul 15;10(7):e0132995. doi: 10.1371/journal.pone.0132995 (PMC4503561; doi:10.1371/journal.pone.0132995)
Supplement: S1 Appendix — The document displays our search strategy for the Medline database. The search was adapted accordingly for the Embase and Cochrane databases (DOCX) [file pone.0132995.s002.docx]

**S1 APPENDIX**

search strategy for the pubmed database

| **Search** | **PubMed Query** | **Items found** |
| --- | --- | --- |
| [#15](http://www.ncbi.nlm.nih.gov/pubmed) | Search **#12 AND #13 AND #14** | [1410](http://www.ncbi.nlm.nih.gov/pubmed/?cmd=HistorySearch&querykey=15) |
| [#14](http://www.ncbi.nlm.nih.gov/pubmed) | Search **("Diagnosis"[Mesh] OR "diagnosis" [Subheading] OR diagnosis[tiab] OR diagnostic[tiab] OR predict[tiab] OR prediction[tiab] OR "Biological Markers"[Mesh] OR biomarker[tiab] OR biomarkers[tiab] OR marker[tiab])** | [8696347](http://www.ncbi.nlm.nih.gov/pubmed/?cmd=HistorySearch&querykey=14) |
| [#13](http://www.ncbi.nlm.nih.gov/pubmed) | Search **("gastrointestinal tract"[MeSH:noexp] OR "Upper gastrointestinal tract"[MeSH:noexp] OR "lower gastrointestinal tract"[MeSH:noexp] OR "digestive system"[MeSH:noexp] OR "digestive system surgical procedure"[MeSH:noexp] OR colon[MeSH:noexp] OR colonic[tiab] OR stomach[MeSH:noexp] OR stomach[tiab] OR rectal[tiab] OR rectum[MeSH Terms] OR "colorectal surgery"[MeSH:noexp] OR duodenum[MeSH:noexp] OR duodenal[tiab] OR pancreas[MeSH:noexp] OR pancreatic[tiab] OR liver[MeSH:noexp] OR hepatic[tiab])** | [960845](http://www.ncbi.nlm.nih.gov/pubmed/?cmd=HistorySearch&querykey=13) |
| [#12](http://www.ncbi.nlm.nih.gov/pubmed) | Search **(CRP[tiab] OR "C-reactive protein"[MeSH Terms]) AND (surgery[MeSH:noexp] OR surgery[tiab] OR surgical[tiab] OR "Anastomosis, Surgical"[Mesh] OR anastomoses[tiab] OR anastomosis[tiab] OR "Ostomy"[Mesh] OR ostomy[tiab] OR ostomies[tiab]))** | [3329](http://www.ncbi.nlm.nih.gov/pubmed/?cmd=HistorySearch&querykey=12) |
